# Designing evidence-based support aids for social media access for individuals with moderate-severe traumatic brain injury: A preliminary acceptability study

**DOI:** 10.3389/fdgth.2022.991814

**Published:** 2022-12-20

**Authors:** Fangyun Zhao, Hajin Lim, Emily L. Morrow, Lyn S. Turkstra, Melissa C. Duff, Bilge Mutlu

**Affiliations:** ^1^ Department of Computer Sciences, University of Wisconsin-Madison, Madison, WI, United States; ^2^ Department of Psychology, University of Wisconsin-Madison, Madison, WI, United States; ^3^ Department of Hearing & Speech Sciences, Vanderbilt University Medical Center, Nashville, TN, United States; ^4^ School of Rehabilitation Science, McMaster University, Hamilton, ON, United States

**Keywords:** technology aids, social media, traumatic brain injury, social participation, accessibility

## Abstract

**Background:**

Adults with traumatic brain injury (TBI) report significant barriers to using current social media platforms, including cognitive overload and challenges in interpreting social cues. Rehabilitation providers may be tasked with helping to address these barriers.

**Objectives:**

To develop technological supports to increase social media accessibility for people with TBI-related cognitive impairments and to obtain preliminary data on the perceived acceptability, ease of use, and utility of proposed technology aids.

**Methods:**

We identified four major barriers to social media use among individuals with TBI: sensory overload, memory impairments, misreading of social cues, and a lack of confidence to actively engage on social media platforms. We describe the process of developing prototypes of support aids aimed at reducing these specific social media barriers. We created mock-ups of these prototypes and asked 46 community-dwelling adults with TBI (24 females) to rate the proposed aids in terms of their acceptability, ease of use, and utility.

**Results:**

Across all aids, nearly one-third of respondents agreed they would use the proposed aids frequently, and the majority of respondents rated the proposed aids as easy to use. Respondents indicated that they would be more likely to use the memory and post-writing aids than the attention and social cue interpretation aids.

**Conclusions:**

Findings provide initial support for social-media-specific technology aids to support social media access and social participation for adults with TBI. Results from this study have design implications for future development of evidence-based social media support aids. Future work should develop and deploy such aids and investigate user experience.

## Introduction

The prevalence of social media and computer-mediated communication (CMC) platforms have altered how people establish social connections, engage in social events, obtain information, and maintain effective collaboration in daily life (1–3). A growing body of research shows that engagement in social media and CMC, particularly *via* Facebook, increases social connectedness and decreases loneliness, plays a critical role in friendship maintenance, and promotes health and well-being (4, 5). For individuals with health-related concerns, social media platforms have provided an important mechanism to find health information, participate in support groups, and share their experiences (6, 7). Individuals with traumatic brain injury (TBI) may particularly benefit from social media, given that they often report social isolation (8) and friendship loss (9), along with physical and cognitive limitations that make in-person social interactions difficult (10, 11). Previous research suggested that social media can promote mental well-being among individuals with TBI and allow them to keep or increase opportunities for social participation (12, 13). Individuals with TBI want to use social media platforms such as Facebook and Twitter as much as their uninjured peers (14). However, these individuals may experience cognitive impairments and have reported significant barriers to using current social media platforms, including cognitive overload and challenges in interpreting social cues (12, 14–17), so the potential benefits of social media are often not accessible to them. Rehabilitation professionals see social media use as a way to reduce social isolation following brain injury, and such professionals may play a future role in addressing barriers to increase social media participation (18).

Social media platforms have provided limited support for increasing accessibility to individuals with TBI and other populations with cognitive impairments (19–21). Accessibility features of social media platforms mostly focus on supporting individuals with sensory disabilities such as hearing or vision impairments (22–24). These features include allowing voice-over gestures for navigating social media sites and providing automatically generated image captions (22, 25). There are no parallel supports for individuals with cognitive impairments such as those routinely observed in individuals with TBI.

The current study is part of a broader effort to develop technological supports to increase social media accessibility for people with TBI-related cognitive impairments. A long-term goal of this line of work is to also understand individual differences that may influence who is willing to use, and who would benefit from, technological support to increase social media accessibility. Here, we report on the process of designing four social media support aids that address challenges in using social media platforms associated with social and cognitive impairments in adults with TBI reported in the literature and those we have observed in the clinical experiences of the author team. The future success of any technological support to improve accessibility and social media use, however, depends on their acceptability and perceived utility and benefit to individuals with TBI (26). Thus, as a first step in this process, we obtained preliminary feedback from individuals with TBI on the acceptability and potential use of these aids to guide future development.

In the following sections, we review previous literature on social and cognitive impairments in individuals with moderate-severe TBI that would affect use of social media platforms and identify four main barriers. We describe potential technological support aids to address these barriers and the process of designing prototypes of these aids. Finally, we report on a survey study where we presented mock-ups of these aids to gain acceptability data and perceptions of the utility of the aids.

## Background

### Social and cognitive impairments in individuals with TBI

Individuals with TBI have a range of deficits that make it difficult to navigate the social world. Impairments in social communication skills are a hallmark of TBI, including impairments in recognizing and interpreting social cues (14, 15, 27–29); missing implied meanings such as sarcasm and jokes; and losing track of topics in a conversation (30–33). These social communication deficits are thought to be a major contributor to the negative social outcomes reported by many adults with TBI (34–36). Indeed, as a group, adults with TBI report having fewer friends and social contacts overall (19), and less social participation with, and more social isolation from, their uninjured peers (20). These negative outcomes in turn affect mental health and wellbeing, not only for the person with TBI but also for their caregivers (37, 38). Impairments in basic cognitive functions are also common following TBI in domains such memory (39), attention (40, 41), decision-making (42–44), and executive functioning (45, 46). These social communication and cognitive deficits have typically been examined and reported in face-to-face, in-person interactions, but recent work suggests that they might extend to computer-mediated communication on social media platforms (18, 47).

### Four evidence-based social media barriers among individuals with TBI

The literature on barriers and challenges to social media use among individuals with TBI, together with the clinical experiences of some of our team members, identifies four major barriers to social media use among individuals with TBI: sensory overload, memory impairments, misreading of social cues, and a lack of confidence to actively engage on social media platforms (14, 47, 48).

#### Sensory overload

Social media platforms can place high demands on sensory processing and attention. Individuals with TBI report difficulty navigating social media sites, keeping up with rapid feeds, and managing sensory overload (12, 17, 27). Some individuals report going through a try-and-fail process to get familiar with the social media platforms due to lack of instructions (14, 15, 27), being overwhelmed, and going offline. In one study, individuals with TBI reported that they found the information on Twitter meaningless and random due to information overload (14). Difficulty managing attention and disrupted information processing are well documented challenges in face-to-face interactions for individuals with TBI (39, 49, 50). These challenges are consistent with the reports of being overwhelmed and overloaded and ultimately abandoning online sessions. A potential solution to this challenge might include restricting the amount of content displayed at any given time by, for example, discretizing the information that is shown in the form of an “infinite scroll” that is widely used by social media platforms.

#### Memory impairments

Social media platforms can place high demands on working and declarative long-term memory. Social media users must quickly identify the owner of the message or post and recall previous events and histories to interpret a given message, as well as quickly integrate and update memory as new information becomes available. Working and declarative memory impairments commonly follow TBI, and these deficits are likely to pose a challenge for using social media platforms. Indeed, declarative memory impairments affected how individuals with TBI process information on social media (47) and decreased their social media use (17). Providing memory assistance that consolidates previous messages to facilitate comprehension of a current message may help individuals with TBI manage the memory demands of using social media.

#### Misreading of social cues

Computer mediated communication requires users to read social cues from a variety of single and integrated sources including faces, videos, text, and emoji. Deficits in reading social cues in individuals with TBI are well documented. Individuals with TBI have difficulty reading cues in social interaction and managing turn taking (14, 15) and, relative to uninjured peers, are less accurate in facial affect recognition (51, 52) and less sensitive to text-based social cues (53). Such deficits in social communication are consistent with reports of individuals with TBI misreading social cues in social media and experiencing negative consequences (12, 17). Providing users with information about the general sentiment of a post might help individuals with TBI in reading social cues on social media platforms.

#### Lack of confidence to actively engage on social media platforms

Individuals with TBI reported a lack of confidence in engaging in online social activities on social media platforms. They also report using Facebook more passively than actively, i.e., being less likely to post status updates or send direct messages to others on social media compared to uninjured peers (17, 20). In particular, individuals with TBI reported worrying about misreading conversations or making mistakes (17, 28). Support tools that allow individuals with TBI to monitor their messages and get feedback before posting could increase confidence when engaging on social media. If so, increased confidence may result in more active participation, which could in turn provide more opportunities to experience the benefits of social media use reported by neurotypical individuals.

Guided by the literature described above, we designed four aids to address: (1) sensory overload, (2) memory impairments, (3) misreading social cues, and (4) a lack of confidence to actively engage on social media platforms. Our overarching strategy was to design aids that reduced the cognitive or sensory load (e.g., memory) or that provided assistance in meeting the social or cognitive demands (e.g., reading social cues) reported by individuals with TBI as barriers to CMC and that the use of the aids would be as simple and intuitive as possible.

After the initial conceptual design, we engaged in ideation and iterative design to develop specific interface solutions that can be implemented as interface augmentations. We ensured that our designs were technologically feasible using available user interface software (e.g., react.js) and commercial text and visual analysis toolbox (e.g., Watson Natural language understanding, IBM Visual Insights) for future implementation. We then created a mock-up of each design for acceptability testing of the concepts of these aids. The mock-ups were created by capturing screenshots of the Facebook interface and modifying its visual elements to represent the design of our aids.

## Design of social media aids for Facebook

We created the mock-ups for Facebook's platform, as adults with TBI cited it as their most commonly used platform (21, 54). In this section, we present the design rationale and development for the social media aids.

### Attention aids

**Figure 1:**
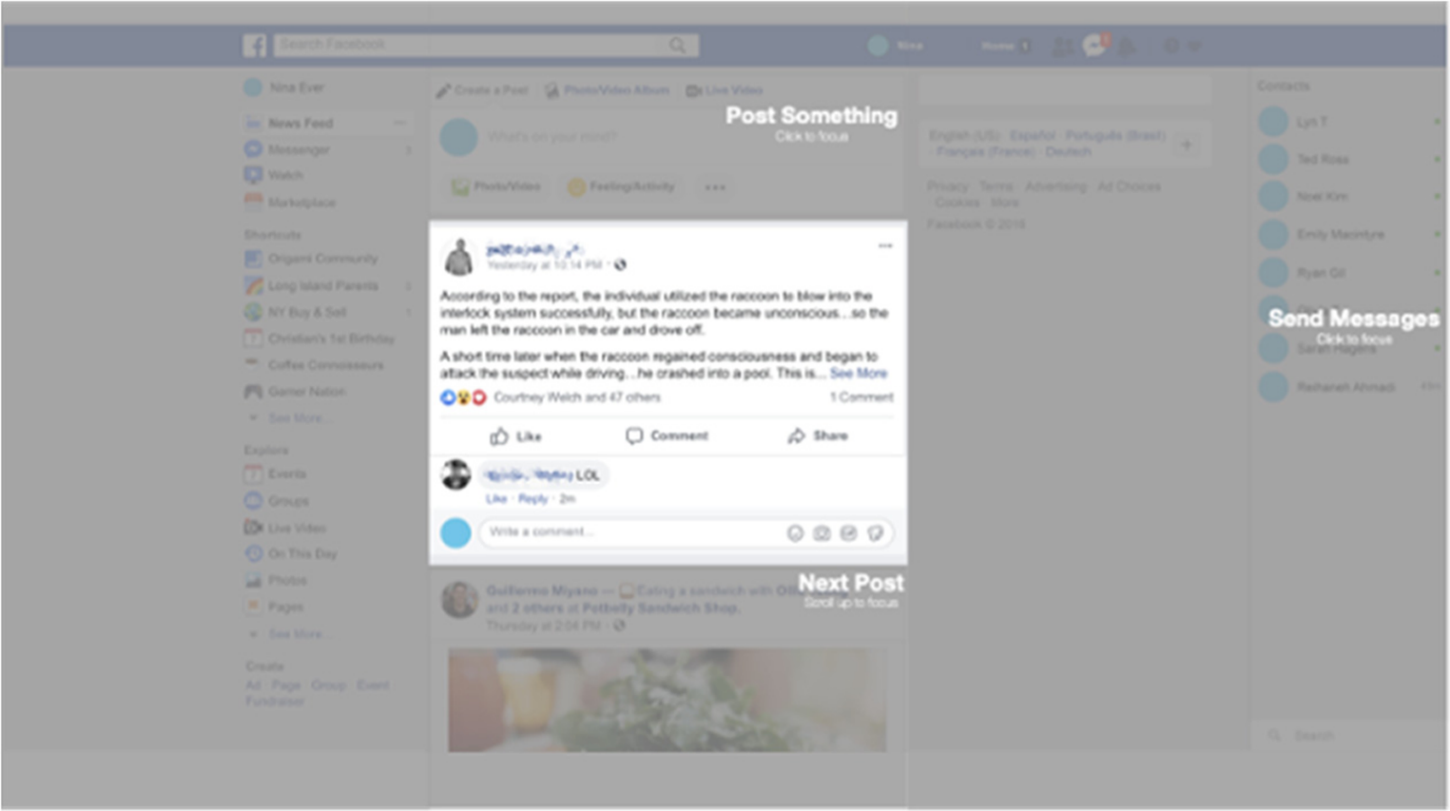
An example of modified Facebook page after using the attention aid. Identifying information such as names and faces have been blurred to protect confidentiality.

To address sensory and information overload reported by individuals with TBI, we aimed to reduce the visual and technical complexity in the current Facebook interface (15, 54) (Figure 1). A traditional Facebook page contains many elements including color side bars, newsfeed posts, friend lists, and advertisements. Arfaa and Wang proposed that grouping and highlighting necessary information could facilitate easier navigation of a website layout for older adults (24). We expected that their suggestion would also be helpful for adults with TBI.

In conceptualizing this aid, we first aimed to reduce visual complexity by putting a transparent gray overlay over the Facebook newsfeed, so users can pay attention to and read one post at a time. Also, to guide better navigation of Facebook, we grouped and labeled each area of a page by its primary purpose (i.e., post status updates, send messages) (see Figure 1). We also created a “Next Post” button to enable users to bring the next post to focus.

### Memory aids

**Figure 2:**
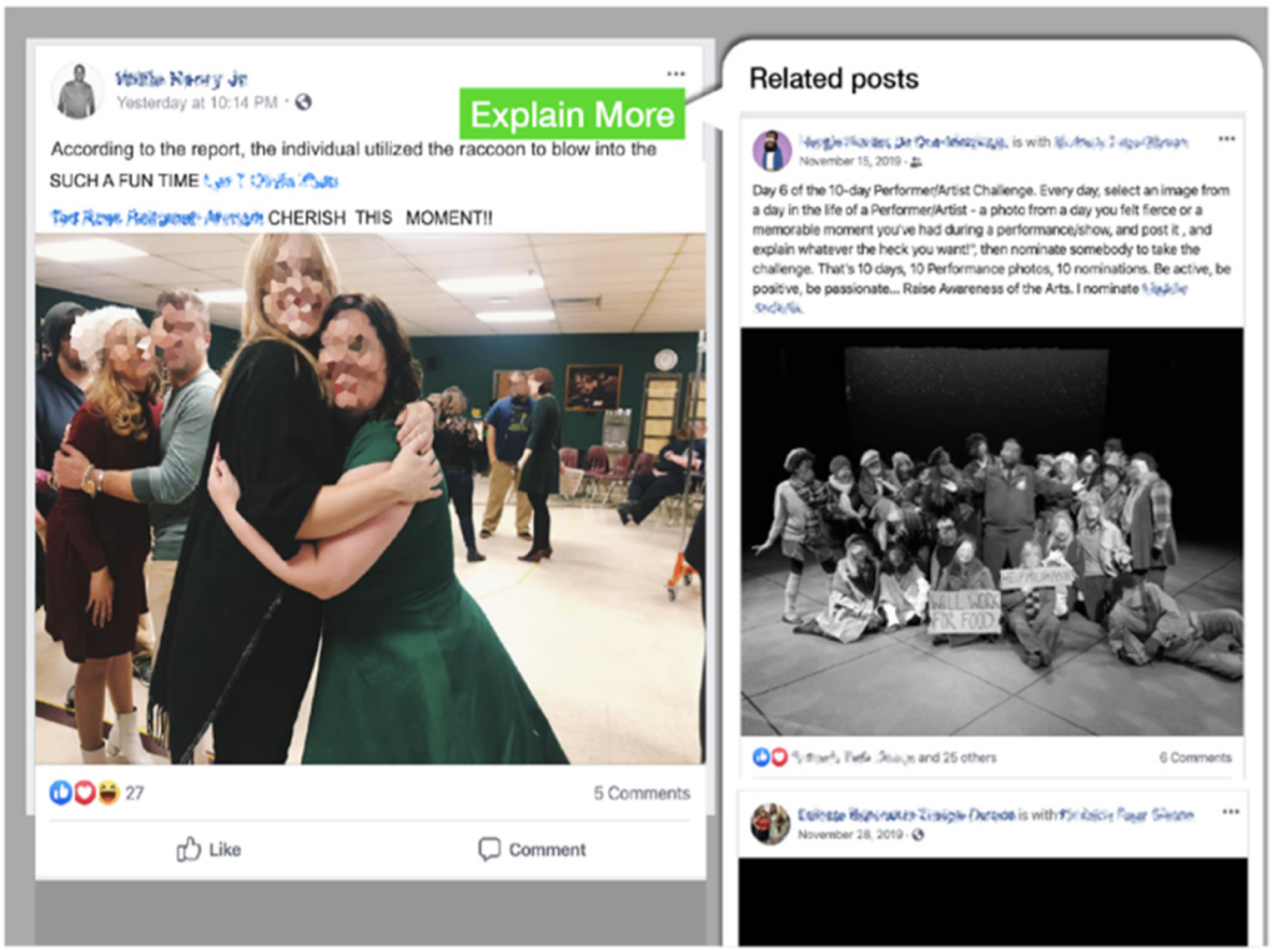
An example of the modified Facebook page using the memory aid. Identifying information such as names and faces have been blurred to protect confidentiality.

To address impairments in memory, we designed an aid that automatically searches and consolidates related posts from a user's profile and presents them to the user. When users see a post that builds on context or information from previous posts, the memory aid retrieves related posts and presents them in a section, titled “explain more,” so users have context for the current post. For example, as illustrated in Figure 2, previous posts about a stage performance were combined into a thread in the “explain more” button. A user could then see how the current post related to previous posts, which provides information implied in the current post and thus supports understanding of that post based on context that may be missing for individuals with a memory deficit (i.e., that it refers to a celebration for the performance).

### Social cue interpretation aids

**Figure 3:**
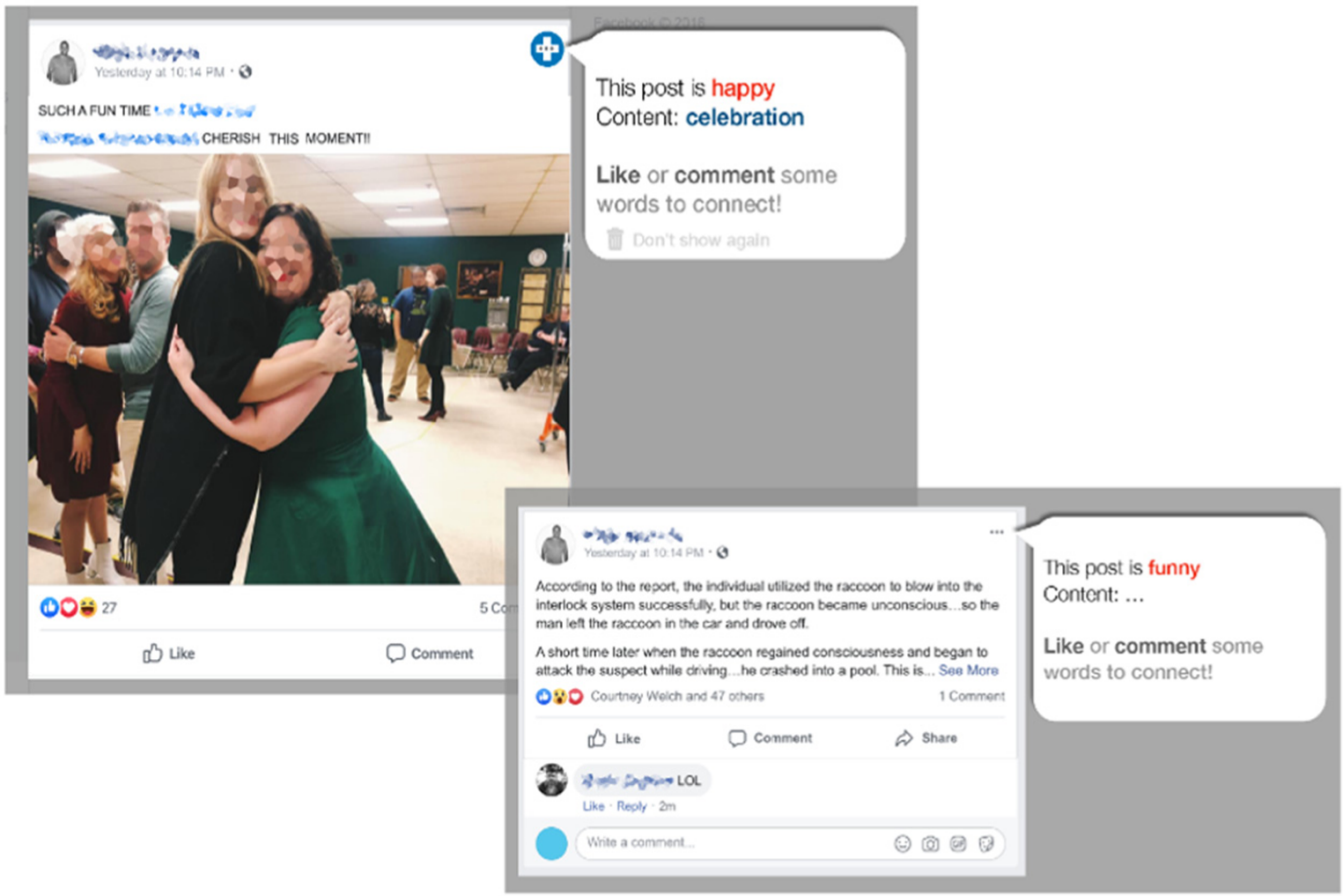
An example of the modified Facebook page using the social cue aid. Identifying information such as names and faces have been blurred to protect confidentiality.

Based on evidence of impaired social communication and misreading of social cues in adults with TBI, we designed an aid to facilitate social cue interpretation. We suspect that reading social cues may be particularly difficult when the demands include integrating the information from text and images. As illustrated in Figure 3, the social cue interpretation aid automatically extracts the main sentiment and topic from the text and/or image from the target post and presents a short summary of the post.

### Message production aids

**Figure 4:**
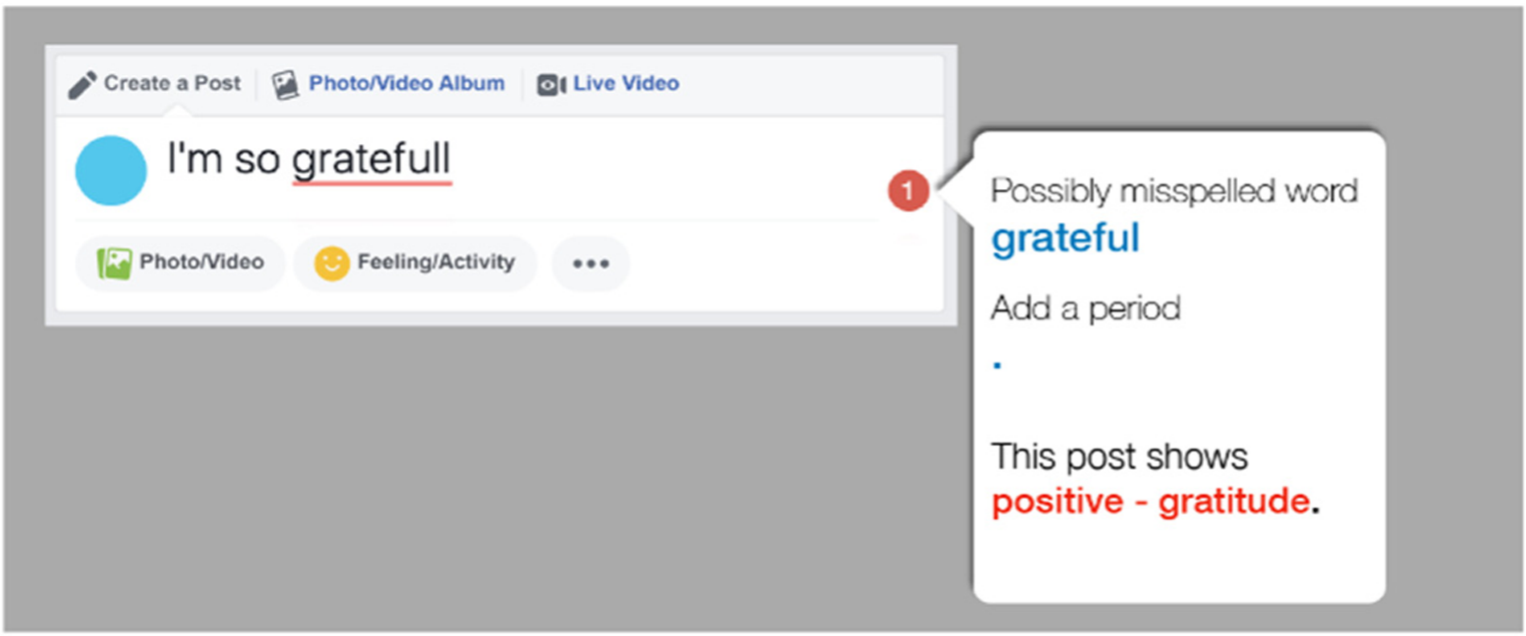
An example of the modified Facebook page using the message production aid.

Individuals with TBI report worrying about misreading conversations and then making mistakes (17, 28). As a consequence, they report being less active on social media. To address this barrier, we designed a message production aid. In addition to providing feedback on spelling and grammar, we expected that this tool would serve as a “Theory-of-Mind check;” that is, it provides feedback on how a recipient would likely interpret that message. As shown in Figure 4, the aid provides feedback on grammar and sentiment of the message before a user posts it, which would give users opportunities to fix grammatical errors and monitor the tone and emotion conveyed by their posts before they are sent. When no error is detected, the system provides positive encouragement to generate their content.

In summary, we identified four key barriers to social media use for individuals with TBI and designed a set of aids aimed at reducing these barriers. Each of these aids included specific design features and functionality to address a key social-cognitive barrier to social media use by individuals with TBI, as summarized in Table 1. Before beginning software development and implementation of the social media support aids, we created mock-ups of our designs to determine if adults with TBI would find these tools acceptable and to obtain additional design suggestions for future social media support tools for individuals with TBI. To obtain this information, we conducted a survey to gauge the acceptability of these aids and perceived use and benefit to individuals with TBI.

**Table 1:** Summary of four aids and their design features.

Type of Aid	Target Barrier	Design Goal	Design Features
Attention Aids	Sensory overload	Reduce visual complexity	A semi-transparent overlay to cover the page; a summary of the functions of each area; highlighting of targeted area of interest
Memory Aids	Memory impairments	Consolidate and present previous posts to help users comprehend the current post	An added button that retrieves related posts
Social Cue Interpretation Aids	Misreading of social cues	Provide a summary of the sentiment of targeted post	An added button that shows the topic and the sentiment of the current message
Message Production Aids	Lack of confidence in actively engaging on social media platforms	Reduce grammatical errors; provide a preview of the sentiment of the message	A button that indicates errors in the current message being produced; when clicked, a message appears that includes errors, a suggested fix, and the sentiment of the current message

## Methods for acceptability study

### Participants

Participants were recruited through the Vanderbilt Brain Injury Patient Registry (55) and were a subset of individuals with TBI surveyed by Morrow and colleagues (21; see below). The study by Morrow et al. included 53 adults (28 females) with moderate-severe TBI, but we excluded seven participants from that sample who reported that they never had a Facebook account. The final sample was 46 participants with moderate-severe TBI (24 females, *M* = 38.0 years old, SD = 9.6). Participants with TBI had 14.9 years of education (SD = 2.3), on average.

All participants with TBI were in the chronic phase of injury (>6 months post-injury) and sustained their injuries in adulthood (i.e., after age 18). Thus, participants’ neuropsychological profiles were in the chronic and stable phase (56). Average time since injury was 71.8 months (SD = 64.0). Participants with TBI did not have a history of neurological or cognitive disabilities before the qualifying brain injury. TBI severity was determined using the Mayo Classification System (57). Participants were classified as having sustained a moderate-severe TBI if at least one of the following criteria was met: (1) Glasgow Coma Scale (GCS) < 13 within 24 h of acute care admission (i.e., moderate or severe injury according to the GCS); (2) positive neuroimaging findings (acute CT findings, or lesions visible on a chronic MRI); (3) loss of consciousness (LOC) > 30 min; or (4) post-traumatic amnesia (PTA) > 24 h. Injury-related information was collected from available medical records and a semi-structured interview with participants.

GCS was available for 38 participants (ranging from 3 to 15); loss of consciousness (LOC) information was available for 42 participants; PTA information was available for 44 participants; acute imaging information was available for 44 participants (43 with positive findings). Causes of injury were motor vehicle accidents (25), falls (6), motorcycle or snowmobile accidents (4), being hit by a car as a pedestrian (4), assault (3), non-motorized vehicle accidents (1), being hit by a moving object (1), or other (3).

### Survey & procedures

The data for the acceptability study were collected as part of a larger project investigating social media use among individuals with TBI. Participants received a link to complete the survey online *via* the Research Electronic Data Capture System (REDCap; 29). The full survey consisted of up to 280 questions, depending on participants' responses. Participants with TBI first answered questions related to their general social media use (reported in ref. 21), how their Facebook usage changed after the injury, their current experience with Facebook, and their perceived social support and social connectedness on Facebook. The results reported here were from the second part of the survey, in which we presented the mock-up images of the prototype designs for the four aids, with explanations of their features, and asked participants about their perceptions of each prototype design. That is, participants were presented with screen shot images to give the sense of the visual appearance and functionality of the aids, but participants could not click on or interact with the aids during this phase of testing.

### Measure

For each aid, participants were asked to complete a 10-item questionnaire. The first five items were from the System Usability Scale (SUS; 58), which has widely been used as a reliable method to measure usability of software products. Aids were referred to as “modifications.” We also modified the wording for question five to make it more relevant to the current study. SUS items were: #1: *I would use this modification frequently*; #2: *I found this modification unnecessarily complex*; *#3: This modification looks easy to use*; #4: *I would need technical support to use this modification;* #5: *Most people with TBI would learn to use this modification very quickly*.

The five subsequent items asked participants to rate the perceived benefits of each aid, particularly how that aid could help them more actively engage in Facebook social interaction. The items were: #6: *I would post and/or share more things with this modification;* #7: *I would click on the content shared by my friends more with this modification*; *#8: I would comment more with this modification*; #9: *I would spend more time on Facebook with this modification*; #10: *I would send more messages to my friends with this modification*.

Participants were asked to indicate how much they agreed or disagreed with each of the 10 statements using a three-item scale (Disagree, Neither agree nor disagree, Agree). In addition, participants were asked whether they noticed any changes in the way they used Facebook after brain injury by answering either “yes” or “no.” Participants also answered two open-ended questions regarding their changes in Facebook use since their injury and their recommendations for modifications to the existing Facebook platform.

### Data analysis

The goal of this study was to explore how individuals with TBI perceive the aids we designed to address their reported challenges in using social media. Consistent with this exploratory goal, we primarily used descriptive statistics to analyze participants' responses. We expect that findings would serve as the foundation for future hypothesis-driven research on technology-based social media interventions for individuals with TBI (59). Consistent with this goal, we also performed *ad hoc* exploratory analyses to investigate if individual characteristics such as age, sex, or education influenced the ratings of the aids.

## Results

Responding to individual questions was voluntary, thus, not all participants answered all questions. The number of individuals who responded to a given question is listed in parentheses.

### Overall attitudes towards the aids

Before examining the participants' responses for each aid type separately, we first summed responses for all aids together (Table 2). Overall, 29.7% of respondents agreed that they would use the proposed aids frequently; 33.5% were neutral; and 36.8% disagreed. Most respondents agreed that the aids looked easy to use (59.2%) and that they would not require any technical support (69.2%). Only 24.0% of respondents indicated that the aids appeared unnecessarily complex, and 10.9% indicated that they would struggle to learn how to use them.

**Table 2:** Summary of participants’ responses for all types of aids.

#	Item	Agree % (count)	Disagree % (count)	Neutral % (count)	Total % (count)
1	I would use this modification frequently.	29.7% (54)	36.8% (67)	33.5% (61)	100% (182)
2	I find this modification unnecessarily complex. (Reversed)	38.8% (71)	24.0% (44)	37.2% (68)	100% (183)
3	This modification looks easy to use.	59.2% (109)	10.9% (20)	29.9% (55)	100% (184)
4	I would need technical support to use this modification. (Reversed)	69.2% (126)	5.5% (10)	25.3% (46)	100% (182)
5	Most people with TBI would learn to use this modification very quickly.	39.6% (72)	11.5% (21)	48.9% (89)	100% (182)
6	I would post and/or share more things with this modification.	14.8% (27)	38.5% (70)	46.7% (85)	100% (182)
7	I would comment more with this modification.	14.4% (26)	40.3% (73)	45.3% (82)	100% (181)
8	I would spend more time on Facebook with this modification.	11.6% (21)	46.4% (84)	42.0% (76)	100% (181)
9	I would send more messages to my friends with this modification.	14.4% (26)	42.2% (76)	43.3% (78)	100% (180)
10	I would click on the content shared by my friends more on this modification.	21.5% (39)	33.7% (61)	44.8% (81)	100% (181)

Note: % is the percentage of respondents who endorsed a statement. Count is the number of respondents who endorsed a statement. Total count is the total number of respondents who answered a given item. The variability in total count reflects that not all respondents answered all questions. Maximum total count is 184 (46 respondents and four aid types).

In regard to Facebook functions, 11.6% of respondents agreed that the proposed aids would help them become more active on Facebook; 14.8% agreed they would post more, 14.4% agreed they would comment more; 14.4% agreed they would send more messages to friends; and 21.5% agreed they would click on others' content more often if using the proposed aids. The remaining responses were relatively equally divided between “neutral” and “disagree.”

### Attitudes towards the support aids by type

For the five survey questions about how the aids might affect participants' Facebook use (i.e., spending time on Facebook, posting, commenting, messaging, clicking on content), responses that were not “agree” were largely divided between “neutral” and “disagree.” Thus, in the interest of clarity and brevity, the results for each tool presented in the text include only the percent that agreed with the statement. Percentages in the other two categories are listed in the tables for each aid.

### Attitudes towards the attention aid

In regard to ease of use, 19.6% agreed that they would use the attention aid often; 26.1% agreed that they found it unnecessarily complex; 54.3% agreed that it would be easy to use; 6.5% agreed they would need technical assistance; and 45.5% agreed that people with TBI would learn to use the tool quickly. In regard to Facebook functions, 6.5% agreed that they would spend more time on Facebook if they used the tool; 8.7% agreed that they would post or share more; 8.7% agreed that they would comment more; 6.8% agreed that they would send more messages to friends, and 17.8% agreed that they would click on others' content more. Results are summarized in Table 3.

**Table 3:** Summary of participants’ responses for attention aid.

#	Item	Agree % (count)	Disagree % (count)	Neutral % (count)	Total % (count)
1	I would use this modification frequently.	19.6% (9)	37.0% (17)	43.5% (20)	100.0% (46)
2	I find this modification unnecessarily complex. (Reversed)	30.4% (14)	26.1% (12)	43.5% (20)	100.0% (46)
3	This modification looks easy to use.	54.3% (25)	10.9% (5)	34.8% (16)	100.0% (46)
4	I would need technical support to use this modification. (Reversed)	60.9% (28)	6.5% (3)	32.6% (15)	100.0% (46)
5	Most people with TBI would learn to use this modification very quickly.	45.5% (20)	4.5% (2)	50.0% (22)	100.0% (44)
6	I would post and/or share more things with this modification.	8.7% (4)	41.3% (19)	50.0% (23)	100.0% (46)
7	I would comment more with this modification.	8.7% (4)	43.5% (20)	47.8% (22)	100.0% (46)
8	I would spend more time on Facebook with this modification.	6.5% (3)	45.7% (21)	47.8% (22)	100.0% (46)
9	I would send more messages to my friends with this modification.	6.8% (3)	40.9% (18)	52.3% (23)	100.0% (44)
10	I would click on the content shared by my friends more on this modification.	17.8% (8)	31.1% (14)	51.1% (23)	100.0% (45)

Note: % is the percentage of respondents who endorsed a statement. Count is the number of respondents who endorsed a statement. Total count is the total number of respondents who answered a given item. The variability in total count reflects that not all respondents answered all questions. Maximum total count is 46 (46 respondents).

### Attitudes towards the memory aid

In regard to ease of use, 37.0% of respondents agreed that they would use the memory aid frequently; 28.9% agreed that the memory aid looked complex; 8.7% agreed that it would be difficult to use; 10.9% agreed that they would need technical assistance to use it; and 32.6% agreed that most people with TBI would learn to use it quickly. In regard to Facebook functions, 13.3% agreed that the memory aid could help them spend more time on Facebook; 13.0% agreed that they would post or share more; 8.9% agreed that they would comment more; 13.0% agreed that they would send more messages; and 21.7% agreed that they would click on content shared by others more. Results are summarized in Table 4.

**Table 4:** Summary of participants’ responses for memory aid.

#	Item	Agree % (count)	Disagree % (count)	Neutral % (count)	Total % (count)
1	I would use this modification frequently.	37.0% (17)	32.6% (15)	30.4% (14)	100.0% (46)
2	I find this modification unnecessarily complex. (Reversed)	37.8% (17)	28.9% (13)	33.3% (15)	100.0% (45)
3	This modification looks easy to use.	58.7% (27)	8.7% (4)	32.6% (15)	100.0% (46)
4	I would need technical support to use this modification. (Reversed)	63.0% (29)	10.9% (5)	26.1% (12)	100.0% (46)
5	Most people with TBI would learn to use this modification very quickly.	32.6% (15)	15.2% (7)	52.2% (24)	100.0% (46)
6	I would post and/or share more things with this modification.	13.0% (6)	41.3% (19)	45.7% (21)	100.0% (46)
7	I would comment more with this modification.	8.9% (4)	40.0% (18)	51.1% (23)	100.0% (45)
8	I would spend more time on Facebook with this modification.	13.3% (6)	42.2% (19)	44.4% (20)	100.0% (45)
9	I would send more messages to my friends with this modification.	13.0% (6)	43.5% (20)	43.5% (20)	100.0% (46)
10	I would click on the content shared by my friends more on this modification.	21.7% (10)	39.1% (18)	39.1% (18)	100.0% (46)

Note: % is the percentage of respondents who endorsed a statement. Count is the number of respondents who endorsed a statement. Total count is the total number of respondents who answered a given item. The variability in total count reflects that not all respondents answered all questions. Maximum total count is 46 (46 respondents).

### Attitudes towards the social cue interpretation aid

In regard to ease of use, 22% of participants agreed that they would use the social interpretation aid frequently; 26.1% agreed that it was unnecessarily complex; 17.4% agreed that it would take a long time to learn; 56.5% agreed that it was easy to use; and 75.0% agreed that they would not require technical support to use it. In regard to Facebook functions, 8.9% agreed that they would spend more time on Facebook if they had the social cue aid; 8.9% agreed that they would comment more; 17.8% agreed that they would post or share more; 13.3% agreed that they would send more messages to friends; and 24.4% agreed that they would click on more content by others if they had the aid. Results are summarized in Table 5.

**Table 5:** Summary of participants’ responses for social cue interpretation aid.

#	Item	Agree % (count)	Disagree % (count)	Neutral % (count)	Total % (count)
1	I would use this modification frequently.	22.2% (10)	48.9% (22)	28.9% (13)	100.0% (45)
2	I find this modification unnecessarily complex. (Reversed)	32.6% (15)	26.1% (12)	41.3% (19)	100.0% (46)
3	This modification looks easy to use.	56.5% (26)	13.0% (6)	30.4% (14)	100.0% (46)
4	I would need technical support to use this modification. (Reversed)	75.0% (33)	0.0% (0)	25.0% (11)	100.0% (44)
5	Most people with TBI would learn to use this modification very quickly.	37.0% (17)	17.4% (8)	45.7% (21)	100.0% (46)
6	I would post and/or share more things with this modification.	17.8% (8)	40.0% (18)	42.2% (19)	100.0% (45)
7	I would comment more with this modification.	17.8% (8)	44.4% (20)	37.8% (17)	100.0% (45)
8	I would spend more time on Facebook with this modification.	8.9% (4)	51.1% (23)	40.0% (18)	100.0% (45)
9	I would send more messages to my friends with this modification.	13.3% (6)	48.9% (22)	37.8% (17)	100.0% (45)
10	I would click on the content shared by my friends more on this modification.	24.4% (11)	37.8% (17)	37.8% (17)	100.0% (45)

Note: % is the percentage of respondents who endorsed a statement. Count is the number of respondents who endorsed a statement. Total count is the total number of respondents who answered a given item. The variability in total count reflects that not all respondents answered all questions. Maximum total count is 46 (46 respondents).

### Attitudes towards post-writing aid

In regard to ease of use, 40% agreed that they would use the post-writing aid frequently; 15.2% agreed that the attention aid looked complex; 67.4% agreed that it would be easy to use; and 78.3% agreed that they would not require technical support to use it; and 43.5% agreed that most people with TBI would be able to easily learn to use it. In regard to Facebook functions, 17.8% agreed that they would spend more time on Facebook if they used this tool; 68.9% agreed that they would post more; 22.2% agreed that they would comment more; 24.4% agreed that they would send more messages to friends; and 22.2% agreed that they would click on others' comments more. Results are summarized in Table 6.

**Table 6:** Summary of participants’ responses for post-writing ai**d.**

#	Item	Agree % (count)	Disagree % (count)	Neutral % (count)	Total % (count)
1	I would use this modification frequently.	40.0% (18)	28.9% (13)	31.1% (14)	100.0% (45)
2	I find this modification unnecessarily complex. (Reversed)	54.3% (25)	15.2% (7)	30.4% (14)	100.0% (46)
3	This modification looks easy to use.	67.4% (31)	10.9% (5)	21.7% (10)	100.0% (46)
4	I would need technical support to use this modification (Reversed)	78.3% (36)	4.3% (2)	17.4% (8)	100.0% (46)
5	Most people with TBI would learn to use this modification very quickly.	43.5% (20)	8.7% (4)	47.8% (22)	100.0% (46)
6	I would post and/or share more things with this modification.	20.0% (9)	31.1% (14)	48.9% (22)	100.0% (45)
7	I would comment more with this modification.	22.2% (10)	33.3% (15)	44.4% (20)	100.0% (45)
8	I would spend more time on Facebook with this modification.	17.8% (8)	46.7% (21)	35.6% (16)	100.0% (45)
9	I would send more messages to my friends with this modification.	24.4% (11)	35.6% (16)	40.0% (18)	100.0% (45)
10	I would click on the content shared by my friends more on this modification.	22.2% (10)	26.7% (12)	51.1% (23)	100.0% (45)

Note: % is the percentage of respondents who endorsed a statement. Count is the number of respondents who endorsed a statement. Total count is the total number of respondents who answered a given item. The variability in total count reflects that not all respondents answered all questions. Maximum total count is 46 (46 respondents).

### Ad hoc exploration of individual characteristics and types of aids

We were next interested in whether there were individual characteristics (age, sex, education) that influenced the ratings of the aids. To conduct this *ad hoc* exploratory analysis, we converted the response options to numeric values (i.e., Disagree = −1, Neutral = 0, Agree = 1) and conducted an exploratory factor analysis on data from the ten items. Factor analysis with Varimax rotation indicated the presence of two factors: one corresponding to the potential utility of the aids and another corresponding to ease of use (see Table 7). These factors accounted for 43.9% and 21.9% of the variance, respectively. By averaging the items loading on each factor, we created measures of “potential utility of the aids” (Cronbach's *α* = .91) and “ease of use” (Cronbach's *α* = .70).

**Table 7:** Factor analysis of the ten item measures.

#	Item	Factor 1 (Potential Utility)	Factor 2 (Ease of Use)
1	I would use this modification frequently.	.68	
2	I find this modification unnecessarily complex. (Reversed)		.63
3	This modification looks easy to use.		.81
4	I would need technical support to use this modification. (Reversed)		.75
5	Most people with TBI would learn to use this modification very quickly.		.60
6	I would post and/or share more things with this modification.	.85	
7	I would comment more with this modification.	.86	
8	I would spend more time on Facebook with this modification.	.84	
9	I would send more messages to my friends with this modification.	.87	
10	I would click on the content shared by my friends more on this modification.	.82	

Correlation analyses were conducted to explore relationships among the measures for potential utility and ease-of-use ratings (from the factor analysis) for the four types of aids and individual characteristics such as age, sex, education, time since onset (TSO), and frequency of Facebook use. Results are summarized in Table 8.

**Table 8:** Pearson correlation table among variables.

	(Factor 1) Potential Utility	(Factor 2) Ease of Use
Sex	−.03	−.01
Age	.23**	.27**
Education	−.15*	.14
TSO	.14	.09
Frequency of FB use	.00	−.07
Type of Aid	.09	.11
Potential Utility	1	.40**
Ease of Use	.40**	1

* Correlation is significant at the 0.05 level.

** Correlation is significant at the 0.01 level.

There was no significant correlation between type of aid and either potential utility or ease of use (r = .09,.11 respectively, *p* > .05), so we did not conduct *post hoc* correlational analyses for each aid type separately. Age was significantly correlated with both potential utility and ease of use (*r* = .23,.27 respectively, *p* < .01). Education was significantly, but negatively, correlated with potential utility (*r* = −.15, *p* < .05), but not ease of use (*r* = .14, *p* > .05). Sex, TSO, and frequency of Facebook use were not significantly correlated with either ease of use or potential utility (*r* = −.03, −.01 respectively, *p* > .05).

## Discussion

The goal of this study was to elicit feedback on prototype aids designed to reduce social media access barriers for adults with TBI. The aids were based on evidence of barriers to social media use by adults with TBI (e.g., 12, 15, 17, 47, 54) and known cognitive challenges in this population (e.g., 39–42, 45), including challenges we had discovered in studies leading up to this project (e.g., 51, 60–63). The four key barriers to social media use included cognitive overload, memory impairments, deficits in social cognition and communication, and a lack of confidence to actively engage on social media platforms. We designed prototypes of aids to address these barriers, presented mock-ups of these aids to participants with TBI, and asked participants to rate the aids' potential utility and ease of use. While the proposed aids are unlikely to address all barriers to successful social media use, to our knowledge this was the first evidence-based study to introduce the concept of social media aids for people with TBI. The findings here provide a foundation for future development of technological supports to enable individuals with TBI to fully access and participate on social media platforms.

Across all aids, nearly one-third of respondents agreed that they would use the proposed aids frequently. The majority of respondents also agreed that all of the aids would be easy to use without technical support and that most people with TBI could learn to use them quickly. These are positive findings given the cognitive demands of adopting new technology and known cognitive challenges of individuals with TBI.

Among the four aids, respondents indicated that they would be more likely to use the memory and post-writing aids than the attention and social cue interpretation aids. The post-writing aid was rated by users as the most helpful of the four aids and easiest to use. Brunner and colleagues noted that many individuals with TBI already rely on writing supports such as Grammarly to produce messages on social media (17). That familiarity might have contributed to acceptance of the post-writing aid, as it includes traditional spelling and grammar support.

While the memory aid was rated as potentially useful, about 30% of participants found the user interface unnecessarily complex. As shown in Figure 3, the memory aid consolidated previous posts and presented them to the user all at once, which inadvertently added visual complexity to the interface and increased the amount of information presented at once. Further investigation is needed to evaluate the tradeoff between memory-recovery benefit vs. visual and informational complexity cost associated with such aids.

One potential reason for the low agreement on utility of the attention and social cue interpretation aids is that the mock-ups did not fully convey the aids' functionalities and did not offer the experience of seeing the aid in operation while using Facebook. In the context of the attention aid, as seeing the aid function while being presented a large amount of self-relevant information might be necessary to effectively experience the aid's functioning. In the context of the social cue interpretation aid, users might have to experience the difficulty of understanding or interpreting content to appreciate the potential value of such an aid. A second reason, and one that might underlie many of the results, is that individuals with TBI often underestimate their own cognitive challenges “in the moment,” (64, 65) and thus might not have appreciated that they had challenges that the aids could help overcome. While the memory and post-writing aids were similar to what individuals without TBI might use (e.g., commercial products like Grammarly or smart phone apps)) and thus would have face validity without the individual needing to be aware of their own challenges, the social cue perception aid in particular would have been novel to participants and thus might have seemed unnecessary. In the future, it would be helpful to collect subjective and objective measures of participants' cognitive abilities, including social cognition, to determine if insight into one's deficit is a factor in perceived utility of technology aids.

Consistent with the well documented heterogeneity among people with TBI (e.g., 66), the individual differences in attitudes toward the technology aids that we identified in our survey is reflective of the wide range of challenges, needs, and preferences of individuals with TBI. We argue that rehabilitation professionals will play a key role in personalizing the social media use of each individual based on the unique deficits, use patterns, and preferences. Rehabilitation professionals already report that they see social media use as a way to reduce social isolation following brain injury and may play an important supporting role in addressing social media barriers and participating safely on social media platforms (e.g., avoiding online scams) (18). We envision that rehabilitation professionals may also play a critical future role in helping individuals with TBI determine if they might benefit from the type of social media aids reported here and personalizing the social media use of each individual based on their cognitive profile and social media use goals. Indeed, rehabilitation professionals, including speech-language pathologists, are particularly well positioned to help individuals with TBI understand how cognitive-communication deficits that are present in face-to-face interactions can extend to computer-mediated communication and can provide training on the features and functionality of future social media aids.

Understanding the utility of these aids requires information about which individuals may most benefit from or be most willing to try social media aids. In an attempt to obtain some preliminary data on individual differences, we conducted an *ad hoc* exploratory analysis on the relation between individual demographic characteristics and potential utility and ease of use of the aids. Participant age had a significant positive correlation with perceived ease of use and utility of social media support tools. An individual's ability to adopt new technology decreases with age later in life (67), which might predict a negative correlation of age with ratings, but older adults in the U.S. are as active on Facebook as younger adults (68). The correlation with age merits replication in the future. Finally, despite the unique cognitive-communicative challenges individuals with TBI face in social media use, younger individuals might more readily accept social media platforms as designed, and older users might see themselves more as benefiting from aids that facilitate their use.

Although there is some evidence of a female advantage in social perception skills in adults with TBI (69), we did not find any effect of sex on perceptions of the social media supports in our study. There is evidence that women are more likely to seek help for healthcare-related concerns (70), but to our knowledge there is no evidence that this tendency extends to cognitive supports such as the aids proposed here.

### Future directions

The current study presents several opportunities for future investigation with the proposed aids. First, based on the initial evaluation on different types of aids, we can prioritize the development of post-writing and memory aids over other types of aids. To extend the potential interests and adoption of these aids to individuals with TBI who are less conscious about their social media use after injury, future studies should consider intervention or tests that can raise awareness on one's social media use patterns and TBI symptoms. Second, in the current study, we found that age might have contributed to the acceptability of aids. However, due to the small sample size, we did not find how the effect of age differed with each aid. For example, we suspect that older adults with TBI might show a stronger interest in memory aids than younger adults with TBI. Future research should seek to better understand individual differences in attitudes towards social media aids with a larger study population. The potential utility of such aids can also be assessed in genuine clinical settings where rehabilitation specialists match the set of aids used by each individual to their cognitive profile, personalize these aids to their needs and preferences, and provide the appropriate training in their use. In this way, individuals with TBI would opt in or opt out of specific aids in the same way social media users can select among other display and security features for personalization.

### Limitations

The study described here was an exploratory study that aimed to assess initial acceptance of the proposed social media aids. We conducted an online survey with static images of the design mock-ups. As a result, respondents might not have been able to fully understand the design concept and engage with the potential functionality of the aids. The critical next step, currently underway, is for participants to test and use the aids over time, to see the costs and benefits in real time. The study also was a relatively small sample of 46 individuals with TBI, and thus our results might not be representative of the general TBI population. The general findings, however, were similar to those reported in previous studies, and the sample was similar to those in others studies in regard to age, sex, social media experience, race, and socioeconomic status of participants. Finally, our findings are necessarily shaped by the specific decisions we have made in designing and creating mock-ups of the aids. Iterative improvement, expert feedback, and usability testing of our designs can ensure future aids that are more effective and widely accepted.

## Conclusions

Adults with TBI report significant barriers to using current social media platforms. We are working to develop technological supports to increase social media accessibility for people with TBI-related cognitive impairments. Here, we found initial support for social-media-specific technology aids to support social media access and social participation for adults with TBI. Future work should develop and deploy such aids and investigate user experience. Future work should also investigate the role of rehabilitation providers in personalizing the social media use of each individual based on the unique deficits, use patterns, and preferences.

## Data Availability

The original contributions presented in the study are included in the article/Supplementary Material, further inquiries can be directed to the corresponding author.
